# Symptoms of depression, anxiety and stress in health students and impact on quality of life*

**DOI:** 10.1590/1518-8345.6315.3885

**Published:** 2023-04-17

**Authors:** Pedro Henrique Batista de Freitas, Adriana Lúcia Meireles, Isabely Karoline da Silva Ribeiro, Mery Natali Silva Abreu, Waléria de Paula, Clareci Silva Cardoso

**Affiliations:** 1 Universidade Federal de São João Del-Rei, Divinópolis, MG, Brasil.; 2 Universidade Federal de Ouro Preto, Departamento de Nutrição Clínica e Social, Ouro Preto, MG, Brasil.; 3 Universidade Federal de Minas Gerais, Departamento de Gestão em Saúde, Belo Horizonte, MG, Brasil.; 4 Universidade Federal de Ouro Preto, Ouro Preto, MG, Brasil.

**Keywords:** Quality of Life, Students, Health Occupations, Students, Mental Health, Depression, Anxiety, Calidad de Vida, Estudiantes del Área de la Salud, Estudiantes, Salud Mental, Depresión, Ansiedad, Qualidade de Vida, Estudantes de Ciências da Saúde, Estudantes, Saúde Mental, Depressão, Ansiedade

## Abstract

**Objective:**

to evaluate the association between quality of life and presence of symptoms of depression, anxiety, and stress in college students in the health area.

**Method:**

cross-sectional study that included 321 students from undergraduate courses in the health area. Quality of life was measured using the World Health Organization scale, abbreviated version, in the physical, psychological, social relations and environment domains, and symptoms were assessed by the depression, anxiety and stress scale. Multivariate analysis was performed using robust linear regression to evaluate the association between quality of life and symptoms.

**Results:**

a negative association was observed between the quality of life and depression symptoms in all domains, while anxiety symptoms showed a negative association in the environment domain, and stress symptoms had a negative association in the psychological domain. Symptom severity was unfavorably associated with quality of life, that is, the greater the symptom severity, the lower the mean scores in all domains.

**Conclusion:**

symptoms of depression, anxiety, and stress were prevalent and had a negative impact on students’ quality of life, especially in the presence of depressive symptoms. The decrease in scores was significantly associated with the severity of symptoms.

## Abstract (es)

**Objetivo:**

evaluar la asociación entre la calidad de vida y la presencia de síntomas de depresión, ansiedad y estrés en estudiantes universitarios del área de la salud.

**Método:**

estudio transversal que incluyó a 321 estudiantes de carreras del área de la salud. La calidad de vida se midió mediante la escala de la Organización Mundial de la Salud, versión abreviada, en los dominios físico, psicológico, relaciones sociales y ambiente, y los síntomas se evaluaron por la escala de depresión, ansiedad y estrés. Se realizó un análisis multivariante por medio de regresión lineal robusta para evaluar la asociación entre la calidad de vida y los síntomas presentados.

**Resultados:**

se observó una asociación negativa entre la calidad de vida y los síntomas de depresión en todos los dominios, mientras que los síntomas de ansiedad tuvieron una asociación negativa en el dominio ambiente, y los síntomas de estrés tuvieron una asociación negativa en el dominio psicológico. La gravedad de los síntomas se asoció desfavorablemente con la calidad de vida, es decir, cuanto mayor la gravedad de los síntomas, menores las puntuaciones medias en todos los dominios.

**Conclusión:**

los síntomas de depresión, ansiedad y estrés fueron prevalentes e impactaron negativamente en la calidad de vida de los estudiantes, especialmente en presencia de síntomas depresivos. Las puntuaciones disminuidas se asociaron significativamente con la gravedad de los síntomas.

## Abstract (pt)

**Objetivo:**

avaliar a associação entre qualidade de vida e presença de sintomas de depressão, ansiedade e estresse em estudantes universitários da área da saúde.

**Método:**

estudo transversal que incluiu 321 estudantes de cursos de graduação da área da saúde. A qualidade de vida foi mensurada por meio da escala da Organização Mundial da Saúde, versão abreviada, nos domínios físico, psicológico, relações sociais e meio ambiente, e os sintomas avaliados pela escala de depressão, ansiedade e estresse. Foi realizada análise multivariada utilizando regressão linear robusta para avaliar a associação entre qualidade de vida e sintomas apresentados.

**Resultados:**

observou- se associação negativa entre qualidade de vida e sintomas de depressão em todos os domínios, enquanto os sintomas de ansiedade apresentaram associação negativa no domínio meio ambiente, e os sintomas de estresse tiveram associação negativa no domínio psicológico. A gravidade dos sintomas associou-se de forma desfavorável com a qualidade de vida, ou seja, quanto maior a gravidade dos sintomas, menor a média dos escores em todos os domínios.

**Conclusão:**

sintomas de depressão, ansiedade e estresse mostraram-se prevalentes e com impacto negativo na qualidade de vida dos estudantes, principalmente na presença de sintomas depressivos. A diminuição dos escores foi significativamente associada à gravidade dos sintomas.

**Highlights:**

**(1)** Screening showed high prevalence of anxiety, stress and depression.

**(2)** Symptom severity was related to poorer quality of life.

**(3)** Depression was the symptom with the greatest impact on quality of life.

**(4)** Contribution to the design of screening strategies in health students.

## Introduction

Quality of life (QoL) is a concept that emerges, in contemporary times, as an important indicator of health conditions and effects of treatments and interventions, based on the individual’s perception of the various aspects of their life within a comprehensive and complex field^(1)^. The QoL of college students is a topic that has been increasingly discussed in health and education, considering the importance that this population assumes in the social and public health context^(2)^.

College students in the health field often experience significant changes when they enter university, especially when they need to move away from their family environment and migrate to other cities, facing a period of transition that can impact their health and QoL^(3-4)^. Some specificities during the academic experience may negatively impact their physical and mental health, such as the high workload in teaching activities, overload of academic tasks, and curricular internships in hospitals and primary care with stressful situations, many of them related to death and the dying process^(5)^.

From this perspective, the insertion in a context of high complexity during academic training may contribute to greater vulnerability in the development of mental disorders, notably stress, anxiety, depression, and suicidal ideation, which significantly affect their academic performance and overall health^(6)^. Moreover, there are indications that health students have a lower QoL when compared to the general population and students from other areas of knowledge and similar age groups, especially in the psychological aspect^(7)^.

The specificities that involve the health education process, associated with other individual factors that negatively influence the mental health of students, such as genetic load and family support network, may be related to the high prevalence of mental disorders in this population, especially depression and anxiety disorders, which can affect more than 30% of these students^(8-10)^. In Brazilian college students, for example, there are indications that depression is one of the main predictors of a worse QoL with a negative impact on academic performance and on their future perspectives both in the professional and personal contexts^(11)^.

In general, it is observed that there is a gap in the literature, especially in Brazil, regarding multicenter studies that assess the relationship between QoL and symptoms of depression, anxiety, and stress in college students in the health area, encompassing different courses, considering that most of these studies are usually limited to a single course or institution^(12-14)^. In this context, most investigations, both national and international, focus on assessing QoL in isolation without elucidating its relationship with aspects of mental health^(11,15)^.

The assessment of QoL and the characteristics associated with health students, especially those related to mental health, may point to indicators for planning interventions and timely care, seeking to avoid future unfavorable outcomes such as suicide^(16)^. Thus, this investigation aims to evaluate the association between QoL and the presence of symptoms of depression, anxiety, and stress in health students.

## Methods

### Study type

This is a cross-sectional study conducted with undergraduate health students from public higher education institutions in Minas Gerais.

### Scenario in which the data collection took place

The study was conducted in three Federal Institutions of Higher Education (FIHEs) in Minas Gerais (MG), Brazil, being: Federal University of São João Del-Rei (Divinópolis and São João del-Rei Campus - UFSJ); Federal University of Ouro Preto (Ouro Preto - UFOP); and Federal University of Triângulo Mineiro (Uberaba- UFTM). This research is part of an epidemiological, multicenter survey, carried out with FIHEs in Minas Gerais, aiming to assess the prevalence of depression, anxiety, and stress among college students^(17)^.

### Time period

The data was collected between the months of May to December 2019.

### Population and sample

To determine the sample composition, an eligible population of 5,847 students from the three educational institutions described was considered. The sample size calculation was performed taking into account the following parameters: 30% estimated prevalence for anxiety and depression disorders^(18)^, a margin of error of 3%, design effect 1.0, and significance level of 5%. The sample size required to meet these parameters was 306 students and was calculated using the OpenEpi ***^®^*** software.

Proportional stratified sampling was used by means of random drawing with replacement, based on the list of enrolled students and the proportional quantity of each university. For the replacement, a quantitative of 30% of students was considered, besides the calculated sample size. Thus, 400 students were contacted.

### Selection criteria

The study population consisted of students from nine undergraduate health courses, over 18 years of age, of both genders, who were regularly enrolled in the three universities at the time of the study. Those enrolled in the following health courses at these institutions were eligible: Biomedicine, Physical Education, Nursing, Pharmacy, Physiotherapy, Medicine, Nutrition, Psychology, and Occupational Therapy. We excluded from the study students who were on exchange at the time of data collection and those who presented repeated answers throughout the questionnaire items. For each student excluded or who did not answer the questionnaire (refusal or non-response), a replacement draw was carried out within the same profile.

### Data collection and instruments used

Data were collected online by means of a virtual, self-applied and confidential questionnaire, made available on the online forms platform “Google Forms” and sent by email to each selected student. Access to this questionnaire was possible by smartphone, tablet or computer.

The participants were previously informed: a) that the average time spent to answer the questionnaire was 30 minutes; b) that it was not mandatory to answer all the questions, being free not to answer those they did not feel comfortable with and; c) about the need to read and sign the Free and Informed Consent Form (FICT), composed of a page explaining the study and requesting authorization to use the data.

To measure QoL, the WHOQOL-bref (World Health Organization Quality of Life Scale) was used, the World Health Organization (WHO) Quality of Life Scale developed by the QoL Group. It is a generic instrument, composed of 26 questions encompassing four domains: physical, psychological, social relations, and environment. The results of the individual domains are scaled in a positive direction, i.e., the higher the score, the better the QoL in the last 15 days. In the validation study for Brazil, this instrument showed satisfactory internal consistency, discriminant validity, criterion validity, concurrent validity, and test-retest reliability^(19)^.

The presence of symptoms of depression, anxiety, and stress was assessed through DASS-21 (Depression, Anxiety and Stress Scale), Depression, Anxiety and Stress Scale adapted and validated for Brazil, presenting adequate characteristics of reliability and validity in the adaptation and validation study^(20)^. It is composed of 21 affirmative sentences, subdivided into three subscales that assess, by self-report, the symptoms of anxiety, depression, and stress during the last week. Each of these subscales is composed of seven questions, and the answers are obtained according to a 4-point Likert scale (0 to 3). The scores for each subscale are obtained by adding the scores of its items and multiplying the total by two. The scores generate the following symptom severity categories: “normal”, “mild/moderate”, severe/ very severe”.

### Study variables

The study variables were: a) QoL indicators measured by WHOQOL-bref scale, defined as a response variable, and b) symptoms of depression, anxiety, and stress assessed by DASS-21 as explanatory variables. Sociodemographic characteristics, lifestyle, and physical health were variables used as adjustment variables for the multivariate model.

### Data treatment and analysis

Data analysis was performed using descriptive and inferential statistics, using R software (version 4.0.5). The results of the QoL evaluation in each domain were transformed into a linear scale from 0 to 100. Descriptive statistics were performed with measures of central tendency and dispersion as well as calculation of relative frequency. In the comparison between QoL and symptoms of depression, stress, and anxiety, Spearman’s correlation coefficient and the Kruskal-Wallis test were used; in multiple comparisons, the Nemenyi.

To evaluate the association between QoL and symptoms of depression, anxiety, and stress, a robust linear regression analysis was performed, considering that the variables did not have a normal distribution. The sociodemographic, clinical, and lifestyle variables that were significant (p<0.20) in the bivariate analysis were used as an adjustment in the multivariate analysis, using the backward method. All explanatory variables were tested for multicollinearity. Missing answers were treated as missing, using the pairwise method.

In the multivariate analysis, depression, anxiety, and stress symptoms were initially included, and then the adjustment variables were included one by one in increasing order of p-value from the bivariate. Covariates with p-value < 0.05 remained in the final model. We used the course variable as an adjustment, regardless of the p-value because of its relevance in other investigations^(3,5,7,11)^.

Thus, a model was built for each QoL domain. For each model, the coefficients (β) for each exposure variable and their respective p-value as well as the coefficient of determination (R^2^) were calculated. The residuals of each model were analyzed for their normal properties. Negative beta (β) values indicate a negative association with QoL scale scores (in units), while positive values indicate a positive association.

### Ethical aspects

This study was approved by the Research Ethics Committee of UFSJ/Campus Centro Oeste (opinion #3,490,510/2019) and by the Research Ethics Committee of UFOP (opinion # 2,621,978/2018). The study participants marked, in a virtual way, their agreement with the free and informed consent form and approved by the Ethics Committee. This research followed all the legal prerogatives of research involving human beings.

## Results

Out of the 400 students who received the invitation to participate in the study, one was on an exchange program and two presented repeated answers throughout the questionnaire items (exclusions) as well as two refused to participate in the study, and 74 did not answer the questionnaire and did not provide a justification. Thus, 321 students from the health area courses participated in the study, distributed among the three institutions: UFOP (28.9%), UFTM (37.0%), and UFSJ (33.9%). Regarding the courses that comprised the sample, the following distribution was obtained: Medicine (27.10%), Pharmacy (15.58%), Psychology (13.08%), Nursing (11.21%), Physical Education (9.66%), Nutrition (10.59%), Physiotherapy (5.61%), Occupational Therapy (3.74%) and Biomedicine (3.43%). Table 1 shows the sociodemographic, clinical, lifestyle, and mental health variables, and it was observed that most were female (71%), single (92.8%), from families with income equal to or higher than 4 minimum wages (38.6%), with a mean age of 24.0 years (±4.04). Regarding lifestyle habits, it was found that most participants reported using alcoholic beverages (72.6%), and excessive use was reported by a considerable portion (47.7%). Almost half (45.5%) reported current or past use of illicit drugs, and a portion reported starting to use them after entering the university (23.7%). The majority reported practicing some physical activity (62.6%). Almost half of the population rated their health as good (49.2%). Severe or very severe symptoms of depression, anxiety, and stress were found in 20.7%, 31.5%, and 23.4% of the students, respectively. The mean BMI was 24.01 (4.04).

**Table 1 -:** Sociodemographic, clinical, life habits and mental health characteristics of health students from three Federal Institutions of Higher Education (n=321). Minas Gerais, Brazil, 2019

**Variables**	**Categories**	**n=321**
		n (%)
**Sociodemographic**		
University 01		93 (28.97)
University 02		109 (33.96)
University 03		119 (37.07)
Major	Medicine	87 (27.1)
	Others	234 (72.9)
Semester	1 and 2	67 (20.87)
	3 to 6	152 (47.35)
	7 to 16	102 (31.78)
Skin color	White	173 (53.89)
	Not white	148 (46.11)
Biological sex	Female	228 (71.03)
	Male	93 (28.97)
Gender identity	Cisgender	318 (99.07)
	Transgender	3 (0.93)
Sexual Orientation	Heterosexual	255 (79.44)
	Homosexual	33 (10.28)
	Others*	33 (10.28)
Marital status^†^	Single	298 (92.83)
	Married	13 (4.05)
	Other^†^	10 (3.12)
Children	Yes	17 (5.3)
Education of the head of the household	Illiterate/Primary	57 (17.76)
	High School	101 (31.46)
	College	163 (50.78)
Income of the head of household	Up to 2 minimum wages	100 (31.15)
	From 2 to 4 minimum wages	97 (30.22)
	More than 4 minimum wages	124 (38.63)
Paid work	Yes	41 (12.77)
University scholarship	Yes	87 (27.1)
Housing	Alone	36 (11.21)
	Parents/relatives	85 (26.48)
	Others^‡^	200 (62.31)
Number of residents in the household	1	41 (12.77)
	2 to 3	159 (49.53)
	4 to 5	75 (23.36)
	6 or more	46 (14.33)
Religious beliefs	Catholic	134 (41.74)
	Evangelical	38 (11.84)
	Others^§^	149 (46.42)
Age	(Average and standard deviation)	24.01 (4.04)
**Life habits**		
Use of alcoholic beverages	Yes	233 (72.59)
Drinking in excess^||^	Yes	134 (47.69)
Smoking	Yes	41 (12.77)
Use of illicit drugs	Has used or uses	146 (45.48)
Drug use after college	Yes	76 (23.68)
Intensified use after college	Yes	44 (15.22)
Physical activity^¶^	Yes	201 (62.62)
**Clinical**		
Family history of depression	Yes	164 (51.09)
Family history of anxiety disorder	Yes	171 (53.44)
Use of CNTD medication^**^	Yes	37 (11.53)
Antidepressant use	Yes	47 (14.64)
Benzodiazepine use	Yes	31 (9.66)
Psychological therapy	Has gone/goes	169 (52,65)
Self-assessment of health	Very good	37 (11.53)
	Good	47 (14.64)
	Regular	31 (9.66)
	Bad	218 (67.91)
BMI^††^	(Average and SD)	23.1 (4.11)
**Mental health**		
Depression Symptoms	Normal	141 (43.93)
	Mild/Moderate	114 (35.51)
	Severe/Very Severe	66 (20.56)
Anxiety symptoms	Normal	149 (46.42)
	Mild/Moderate	71 (22.12)
	Severe/Very Severe	101 (31,46)
Stress symptoms	Normal	110 (34.27)
	Mild/Moderate	136 (42.37)
	Severe/Very Severe	75 (23.36)

* Bisexual, asexual;

† Divorced, stable union, other;

‡ Republic, boarding house, lodging, spouse;

§ Non-belief, spiritualist, oriental Buddhism, Jewish, Afro-Brazilian, other;

|| Total doses taken on a single occasion (total of five doses for men and four for women on a single occasion);

¶ Practicing physical activity refers to exercise or any physical activity, such as sport (e.g., soccer, tennis, running, swimming, etc.);

** CNTD = Chronic Non-Transmissible Chronic Disease;

†† BMI = Body mass index

Table 2 shows the results of the bivariate analysis of the association between QoL and the presence of symptoms of depression, anxiety, and stress. It was observed that the predictor variables (depression, anxiety, and stress) were significantly associated with the QoL outcome in all domains (p-value 0.05).

**Table 2 -:** Bivariate analysis through the robust linear regression model, evaluating the association between quality of life and symptoms of depression, anxiety, and stress according to the domains of the WHOQOL-bref^*^ scale of health students from three Federal Higher Education Institutions (n=321). Minas Gerais, Brazil, 2019

**Symptoms**		**P-value** ^[Table-fn tfn10b]†^			
		**Physical Domain**	**Psychological Domain**	**Social Relationships**	**Environment**
Depression	Normal	-	-	-	-
	Mild/Moderate	0.000	0.000	0.000	0.000
	Severe/Very Severe	0.000	0.000	0.000	0.000
Anxiety	Normal	-	-	-	-
	Mild/Moderate	0.000	0.000	0.049	0.002
	Severe/Very Severe	0.000	0.000	0.000	0.000
Stress	Normal	-	-	-	-
	Mild/Moderate	0.000	0.000	0.010	0.001
	Severe/Very Severe	0.000	0.000	0.000	0.000

* WHOQOL-bref = World Health Organization Quality of Life Scale;

† p-value < 0.05

The correlation between depression, anxiety, and stress symptoms and QoL is presented in Table 3. It is observed that all QoL domains were significantly (p-value < 0.05) and negatively (r < 0) correlated with depression, anxiety, and stress, i.e., the greater the severity of symptoms, the lower the QoL scores tended to be in all domains. The strongest correlation observed (r= -0.68) was between the psychological domain and depression. In contrast, the weakest correlation observed (r = -0.25) was between the social relations domain and anxiety.

**Table 3 -:** Correlation between depression, anxiety and stress and quality of life of health students from three Federal Institutions of Higher Education (n=321) according to the domains of the WHOQOL-bref^*^ scale. Minas Gerais, Brazil, 2019

	**Depression Score r¹** † **(p-value)**	**Anxiety Score r¹** † **(p-value)**	**Stress Score r¹** † **(p-value)**
Physical Domain	-0.54 (0.000)	-0.45 (0.000)	-0.44 (0.000)
Psychological Domain	-0.68 (0.000)	-0.54 (0.000)	-0.56 (0.000)
Social Relationships	-0.34 (0.000)	-0.25 (0.000)	-0.26 (0.000)
Environment	-0.34 (0.000)	-0.31 (0.000)	-0.29 (0.000)

* WHOQOL-bref = World Health Organization Quality of Life Scale;

† r 1 = Spearman’s correlation coefficient

The comparison of QoL scores in the WHOQOL-bref scale according to the severity of symptoms of depression, anxiety and stress is presented in Table 4. A statistically significant difference (p-value <0.001) in QL scores was observed with symptom severity, i.e., the greater the symptom severity, the lower the mean QoL scores in all domains, also presenting the dose-response gradient. The lowest QoL score was found for the psychological domain in the presence of severe or very severe depression (QoL=36.87) and in the presence of severe or very severe stress (QoL=41.28).

**Table 4 -:** Average scores of quality of life by domains of the WHOQOL-bref * scale in the presence of symptoms of depression, anxiety and stress and result of the comparison test in health students from three Federal Institutions of Higher Education (n=321). Minas Gerais, Brazil, 2019

**Scales**	**QoL Domains** †	**Symptoms**	**Number**	**Average** ‡	**SD** §
Depression	Physical	Normal	141	73.02	13.52
		Mild, Moderate	114	61.12	13.26
		Severe, Very Severe	66	51.52	16.23
	Psychological	Normal	141	69.71	12.44
		Mild, Moderate	114	54.39	14.02
		Severe, Very Severe	66	36.87	18.59
	Social Relationships	Normal	141	68.97	18.18
		Mild, Moderate	114	59.36	20.60
		Severe, Very Severe	66	52.02	22.79
	Environment	Normal	141	67.09	13.82
		Mild, Moderate	114	58.96	12.99
		Severe, Very Severe	66	52.89	18.16
Anxiety	Physical	Normal	149	71.48	14.05
		Mild, Moderate	71	62.98	12.51
		Severe, Very Severe	101	54.88	16.83
	Psychological	Normal	149	66.58	15.18
		Mild, Moderate	71	57.69	14.48
		Severe, Very Severe	101	44.02	19.27
	Social Relationships	Normal	149	66.95	19.17
		Mild, Moderate	71	61.15	20.89
		Severe, Very Severe	101	55.53	22.21
	Environment	Normal	149	66.28	13.56
		Mild, Moderate	71	60.52	12.24
		Severe, Very Severe	101	54.46	17.63
Stress	Physical	Normal	110	72.14	13.16
		Mild, Moderate	136	64.42	15.05
		Severe, Very Severe	75	52.90	16.15
	Psychological	Normal	110	68.56	14.12
		Mild, Moderate	136	57.54	16.82
		Severe, Very Severe	75	41.28	17.81
Stress	Social Relationships	Normal	110	67.65	18.23
		Mild, Moderate	136	61.15	21.38
		Severe, Very Severe	75	55.56	22.53
	Environment	Normal	110	66.22	13.04
		Mild, Moderate	136	60.64	14.90
		Severe, Very Severe	75	55.21	17.71

* *WHOQOL-bref* = *World Health Organization Quality of Life Scale*;

† QoL = Quality of life;

‡ Kruskal-Wallis. Multiple comparisons, all p-value < 0.001;

§ SD = Standard deviation

The model adjusted by sociodemographic, clinical, life habits, and course variables is shown in Table 5. It was observed that depression symptoms showed an impact on decreased QoL in all domains of the scale: 1. Physical domain: symptoms of mild/moderate depression (β:-5.99) and of severe/very severe depression (β:-12.26). 2. Psychological domain: symptoms of mild/moderate depression (β:-9.11) and of severe/very severe depression (β:-22.45). 3. Social relations domain: symptoms of mild/moderate depression (β:-6.18) and of severe/very severe depression (β:-12.31). 4. Environment domain: symptoms of mild/moderate depression (β:-4.22) and severe/very severe depression (β:-9.09). In the same vein, severe/very severe stress symptoms (β:-6.47) were associated with decreased QoL scores in the psychological domain, while severe/very severe anxiety symptoms (β:-5.90) had a negative association in the environmental domain. The course variable, used independently of the p-value, had little influence on the model.

**Table 5 -:** Final adjusted model of the association between quality of life and symptoms of depression, anxiety and stress according to the domains of the WHOQOL- bref * scale of health students from three Federal Institutions of Higher Education (n=321). Minas Gerais, Brazil, 2019

**Variables**		**Quality of life domains**							
		**Physical** †		**Psychological** ‡		**Social Relationships** §		**Environment** ||	
		**β ¶**	**Valor-p**	**β ¶**	**Valor-p**	**β ¶**	**Valor-p**	**β ¶**	**P-value**
D**	Normal	-	-	-	-	-	-	-	-
	Mild, Moderate	-5.99	**0.002**	-9.11	**0.000**	-6.18	**0.025**	-4.22	**0.018**
	Severe, Very Severe	-12.26	**0.000**	-22.45	**0.000**	-12.31	**0.002**	-9.09	**0.002**
A††	Normal	-	-	-	-	-	-	-	-
	Mild, Moderate	-2.10	0.267	-0.13	0.944	-1.41	0.662	-3.76	0.051
	Severe, Very Severe	-3.31	0.192	-2.91	0.291	-2.32	0.549	-5.90	**0.015**
S‡‡	Normal	-	-	-	-	-	-	-	-
	Mild, Moderate	0.19	0.923	-2.75	0.120	0.60	0.821	1.11	0.537
	Severe, Very Severe	-4.00	0.151	-6.47	**0.031**	0.78	0.855	2.38	0.432
R²§§		44.27%		51.18%		18.83%		31.47%	

* WHOQOL-bref = World Health Organization Quality of Life Scale;

† Adjustment variables by domains (P-value <0.20 on bivariate): gender, housing, number of household residents, use of CNTD medication, use of benzodiazepines, self-assessment of health, and course;

‡ Adjustment variables by domains (P-value <0.20 on bivariate): education of head of household, use of antidepressants, self-assessment of health, and course;

§ Adjustment variables by domains (P-value <0.20 on bivariate): household head’s income, housing, alcohol use, self-assessment of health, and course;

|| Adjustment variables by domains (P-value <0.20 in the bivariate): household head’s income, self-assessment of health, and course;

¶ β = Indicates standardized β coefficient;

** D = Depression;

†† A = Anxiety;

‡‡ E = Stress;

§§ R² = Coefficient of determination

## Discussion

Symptoms of depression, anxiety, and stress are interconnected phenomena that move between negative affect, emotional discomfort, and physiological changes^(6,20)^. In this investigation, it was observed that approximately 50% of health students presented mild to very severe symptoms of depression, anxiety, and stress, and more than 20% presented severe symptoms of these conditions. Symptoms of depression were associated with lower QoL in all dimensions with greater impact in the psychological domain, whereas symptoms of anxiety were associated with lower QoL in the environmental domain and symptoms of stress in the psychological domain.

The results of this multicenter study show a peculiar scenario since they indicate that students of undergraduate courses in the health area of the institutions surveyed have a high burden of symptoms of depression, anxiety, and stress with potential interference in their QoL. These findings are innovative, considering that the impact and the relationship between mental health and QoL in students of health courses were not well elucidated so far.

Depressive symptoms are considered the psychological predictor most often associated with low QoL observed in undergraduate health students, especially among nursing and medical students in most domains of the WHOQOL-bref^(2,9,11,21)^. This finding was also demonstrated in this investigation, with a decrease in QoL scores in all domains and with an important response dose gradient, since there was a significant reduction in QoL according to the severity of symptoms. In addition, the strongest correlation was found between the psychological domain and depression symptoms, indicating the impact of this mental health condition on students’ QoL. The psychological domain encompasses emotions, body image, and self-esteem, being strictly related to the individual’s perception of himself and his environment^(19)^.

The psychological domain showed the most significant decrease in scores, especially in the presence of severe symptoms of depression. Other investigations involving undergraduate health course students suggested that the psychological domain was decisively impacted in the presence of depressive symptoms with a significant reduction in QoL scores^(14-15,21-22)^. Despite the divergences indicated by the literature, some factors are pointed out as contributors to the development of depressive symptoms and worsening of QoL throughout the health education process, such as the presence of anxiety, stress, female gender, and economic difficulties^(8,16,21,23)^.

The decrease in QoL observed in health students, especially in the psychological domain, is closely related to the high prevalence of depression symptoms in this population, according to some evidence^(8-9,14,16)^. These students experience a higher prevalence of depressive symptoms, especially severe symptoms, when compared to other areas of knowledge, considering the specificities of their training process, such as the challenges and responsibilities that involve caring for human life, including the process of death and dying^(7,24)^. This high burden of depressive symptoms can cause a negative impact on QoL, with complex risk factors related to individual characteristics (personality traits, for example) and the academic environment^(25)^. Consistent with the findings of this study, it is estimated that more than 30% of students in this area, both nationally and internationally, present moderate to severe symptoms of depression^(9-10)^, which may vary according to different geographic regions and age groups, showing an inverse relationship with QoL^(23)^.

This scenario is increasingly alarming within higher education institutions and public health, considering the negative impact of these symptoms on the physical and mental health of students with unfavorable outcomes, especially a significant decrease in academic performance, disruption of social interaction, suicidal ideation, and increased risk of suicide^(6,14)^, which may be reflected in QoL and health services delivery by these future professionals^(2,9,15,22)^. It is believed that the prevalence of these symptoms is at least partially related to the learning environment, considering the overload of teaching activities, pressure for high performance, contact with challenging situations in practice fields, distance from family members, and low self-esteem^(8,14)^. Paradoxically, meta-analysis studies suggested that the learning environment, by itself, does not present itself as the preponderant aspect for the high burden of depressive symptoms in this population, but influences the training process, contributing to several negative impacts on the health and QoL of these students in the medium and long term^(11,18)^.

A high prevalence of anxiety symptoms among students in this study was found, showing mild and moderate symptoms in 21.1% and severe and very severe symptoms in 31.4% with decreased QoL scores, especially in the presence of severe symptoms. This finding can be corroborated by a recent meta-analysis estimating that about one in three medical students presented anxiety symptoms with an overall prevalence of 33.8%, showing a higher prevalence than in the general population, which may substantially impact QoL^(26)^. Another investigation, which included students from health courses, showed that anxiety symptoms were the most prevalent, affecting 74.6% of students with important participation of stressors related to the academic environment in their development^(6)^. In Brazil, the prevalence of anxiety symptoms in health students is also above 50%, contributing to the worsening of QoL^(24,27)^. Although this condition is as debilitating and common as depression, it is still little discussed and diagnosed among college students^(27-29)^.

It was observed that anxiety symptoms were negatively associated with QoL in the environment domain. The environment domain involves issues related to financial resources, safety and security, physical environment, and recreation and leisure opportunities^(19)^. Students with anxiety symptoms usually experience feelings of insecurity, tension, and fear, depriving themselves of activities that promote well-being, which may cause impairment of academic life, difficulties in social interaction, global functional worsening, suicidal thoughts, and depression^(15,17,22)^.

The severity of anxiety symptoms resulted in decreased QoL scores. Despite the gap in the literature regarding this association in health students, there are indications of the negative influence of anxiety on QoL of these students with lower QoL in the presence of moderate and severe symptoms^(15)^. Research conducted with Malaysian medical students showed that those with anxiety symptoms had a significant decrease in QoL in the psychological and environmental domains of the WHOQOL-bref scale^(22)^, a result consistent with the present investigation. Moreover, this relationship is complex because there are other determinants involved in this process, such as individual aspects, lifestyle, sociodemographic and learning environment characteristics, impacting the increased prevalence and severity of symptoms and, consequently, on the QoL of these students^(12,24-25)^.

The presence of stress symptoms was negatively associated with QoL in the psychological domain and a high prevalence of moderate and severe symptoms was evidenced. Stress can be defined as a non-specific state of excessive excitement or tension, resulting from the inefficiency or exhaustion of coping strategies, having a complex and close relationship with depression and anxiety^(30)^. Students who experience high and persistent levels of stress are more susceptible to decreased academic performance, changes in general health status, and the development of major depressive symptoms^(4,25,31)^. Thus, as demonstrated by this investigation, high levels of stress are associated with worse QoL with a greater impact on the psychological domain, causing a significant decrease in scores according to the severity of symptoms^(6,32-33)^.

This condition has been shown to be common among students of undergraduate courses in the health field. A prevalence of stress of 42.3% of mild and moderate symptoms and 23.3% of severe and very severe symptoms was observed, possibly impacting the perception of QoL. Studies carried out with health students both nationally and internationally point out that stress is considered the most prevalent mental health problem among these students, affecting more than half of them^(6,13,18)^. Besides, there are indications that the prevalence of moderate and severe stress symptoms is higher among health students when compared to other areas of knowledge^(32)^. In this study, some sociodemographic aspects, especially female gender and economic class, were used as adjustment variables in the association between symptoms of depression, anxiety and stress, and QoL. These sociodemographic aspects demonstrate a possible influence on the worsening of mental health and QoL in undergraduate health course students. Observational^(12,22,28,34)^, meta-analysis^(8,11)^, and longitudinal^(23,25)^ studies conducted with health students indicated that female students showed a significant decrease in QoL scores in the physical and psychological domains when compared to males. This finding is possibly due to the high frequency of mental disorders in female students^(18,24,27)^. The stresses and difficulties related to economic aspects (income and housing conditions, for example) can also affect the mental health and QoL of these students^(11,21,28)^. In this context, in the present investigation, even adjusting for socioeconomic, demographic, and physical health characteristics, the symptoms of anxiety, depression, and stress were negatively associated with a worse QoL in all dimensions. This study has some limitations that deserve discussion. The first one is related to the cross-sectional design, in which it is not possible to establish causal relations regarding the influence of variables on QoL. Thus, it is suggested the development of longitudinal studies to better elucidate the causal relationships between the factors mentioned and QoL. Another issue is related to the possibility of information bias, because when approaching controversial or sensitive topics, the student may have presented socially acceptable answers, but this fact may have been minimized, considering the non-personal identification and non- mandatory response to the questions. The generalization of the results should also be analyzed with caution since the sample does not correspond to the totality of public educational institutions in the state of São Paulo.

Even considering its limitations, the findings of this study are relevant and present a worrisome context. Health area students showed a high frequency of depression, anxiety, and stress symptoms with negative impact in all QoL domains. Thus, in light of the findings, this study can be useful to managers of public universities by serving as a parameter for planning and implementing strategies for timely identification of students with symptoms of depression, anxiety, and stress and, according to the results of screening, making referrals for treatment and follow-up throughout the training process. Moreover, the findings can provide subsidies for the possible review of curricula and programs of undergraduate health courses, aiming to plan and implement strategies that promote well-being, and resilience and, consequently, improve the QoL of students.

## Conclusion

In this study, the presence of symptoms of depression, anxiety, and stress showed a negative association with QoL. Symptoms of depression were inversely associated with QoL in all domains of QoL, with a significant decrease in scores and a greater impact on the psychological domain. Considering that these conditions can lead to decreased academic performance and social interaction, in addition to a significant decline in mental health and suicide risk, it is expected to contribute to the area of educational management with the design of timely screening strategies and strategies for improving the QoL of this population.
